# Machine learning for small interfering RNAs: a concise review of recent developments

**DOI:** 10.3389/fgene.2023.1226336

**Published:** 2023-07-13

**Authors:** Minhyeok Lee

**Affiliations:** School of Electrical and Electronics Engineering, Chung-Ang University, Seoul, Republic of Korea

**Keywords:** machine learning, small interfering RNA, SiRNA interference, deep learning, bioinformatics, artificial intelligence, artificial neural network

## Abstract

The advent of machine learning and its subsequent integration into small interfering RNA (siRNA) research heralds a new epoch in the field of RNA interference (RNAi). This review emphasizes the urgency and relevance of assimilating the plethora of contributions and advancements in this domain, particularly focusing on the period of 2019–2023. Given the rapid progression of deep learning technologies, our synthesis of recent research is paramount to staying apprised of the state-of-the-art methods being utilized. It not only offers a comprehensive insight into the confluence of machine learning and siRNA but also serves as a beacon, guiding future explorations in this intersectional research field. Our rigorous examination of studies promises a discerning perspective on the contemporary landscape of machine learning applications in siRNA design and function. This review is an effort to foster further discourse and propel academic inquiry in this multifaceted domain.

## 1 Introduction

Enveloped within the expansive discipline of RNA interference (RNAi) (Wilson and Doudna, 2013; Mansoori et al., 2014; Rosa et al., 2018), the integration of machine learning strategies (Walia et al., 2012; Liu, 2019; Petegrosso et al., 2020) in the design and analysis of small interfering RNAs (siRNAs) marks a significant step in the advancement of this field. siRNAs, as vital components of the RNAi pathway, play an indispensable role in post-transcriptional gene silencing, influencing various genetic processes and, by extension, the potential for therapeutic interventions (Reynolds et al., 2004; Kanasty et al., 2013; Resnier et al., 2013; Dana et al., 2017; Tatiparti et al., 2017; Hu et al., 2020). Our review ventures into this rapidly evolving field, providing a detailed narrative of the seminal research contributions that blend the potent capabilities of machine learning with the inherent complexities of siRNA design and function.

Machine learning employs algorithms that improve automatically through experience (Jordan and Mitchell, 2015; Waring et al., 2020; Greener et al., 2022). It is employed across a myriad of applications, ranging from recommendation systems (Batmaz et al., 2019) to autonomous driving Bachute and Subhedar (2021), and now, increasingly in life sciences (Roscher et al., 2020). The unprecedented pace of machine learning advancements accentuates the need for an in-depth review of the most recent studies, ensuring that researchers and practitioners are abreast with state-of-the-art applications in the field.

It is this synthesis of machine learning and siRNA, an emergent and vital topic, that captures our academic interest. As the landscape of machine learning continues to diversify and mature, and siRNA’s influence in genetic research and therapeutic innovation becomes more profound, our review serves as a catalyst for fostering academic dialogue and nurturing exploratory research. Herein, we have carefully investigated studies published between 2019 and 2023 through Web of Science (WoS) using keywords of machine learning and siRNAs. The utilization of the WoS platform stems from its comprehensive incorporation of solely peer-reviewed journal articles of high quality. The specific timeframe of 2019–2023 was chosen not solely based on the quantity of research produced, but also due to the significant advancements in machine learning techniques, particularly deep learning methodologies, during this period.

**FIGURE 1:**
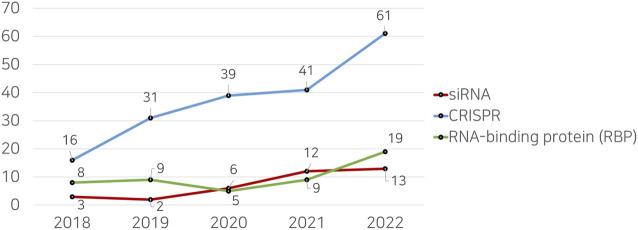
Comparison of the number of research papers retrieved from the Web of Science (WoS) database when searched with the keyword “Machine Learning.”

This academic landscape is mirrored by a discernible gap in research studies focusing on machine learning applications for siRNAs, as shown in Figure 1. The result through the Web of Science database reveals a significantly lesser number of publications on machine learning and siRNAs as compared to other RNA-related topics, such as CRISPR and RNA-binding proteins (RBPs). This indicates an under-explored niche in the application of machine learning methods for siRNA analysis and design. The reviewed studies are summarized in Table 1, weaving together a comprehensive and up-to-date review of this intersectional field.

**TABLE 1:** Overview of recent studies using machine learning methods in siRNA.

Research topics	Brief description	Studies
siRNA Efficacy Prediction	Predictive models built with GNN, ANN, and SVM for siRNA efficacy and targeting	La Rosa et al. (2022), Metwally et al. (2022)
Cancer Research	Identification of prognostic genes and drug release behavior prediction in cancer studies using siRNA and machine learning	Kukita et al. (2022), Kavya et al. (2022)
Cellular Uptake Prediction	Prediction of siRNA nanoparticle uptake in cancer cell lines with Random Forest, Multilayer Perceptron, and Linear Regression models	Nademi et al. (2021)
siRNA Off-Target Effect	Analysis of siRNA off-target effects based on thermodynamic properties of siRNA subregions	Kobayashi et al. (2022)
RNA Production Regulation	Genome-wide siRNA screen to discover global RNA production regulators using supervised machine learning	Müller et al. (2021)
Mitochondrial Dysfunction	Application of machine learning to analyze mitochondrial dysfunction in siRNA-based screening data	Scott et al. (2020)
Gene Delivery	Live-cell analysis device combined with deep learning for gene delivery analysis	Patino et al. (2022)
Proteome Regulation	Machine learning used on proteomics data to explore proteome regulation; validation of new replication factors through siRNA knockdowns	Kustatscher et al. (2023)
Diagnostic Gene Biomarkers	Machine learning used to identify potential diagnostic gene biomarkers with subsequent siRNA knockdown for validation	Sun et al. (2022)
In Silico Drug Discovery	Machine learning integrated with high-content analysis data for *in silico* drug discovery and kinase target identification	Kuthuru et al. (2019)
High-Throughput Screening	High-throughput siRNA knockdown screening with light-sheet microscopy and CNN-based phenotype classification	Eismann et al. (2020)
Nanopore Trapping	Machine learning algorithm developed to aid nanopore trapping/translocation for structural profiling of low molecular weight RNAs	Wang et al. (2021)
vsiRNA Prediction	Prediction model based on deep CNN for identifying virus-derived small interfering RNAs (vsiRNAs)	He et al. (2019)
mRNA Cleavage Site Identification	Deep learning CNN model used to distinguish true mRNA cleavage sites from false ones in the small RNAs (sRNA) landscape	Persson Hoden et al. (2021)

In the course of our comprehensive review of recent developments in the application of machine learning for siRNAs, it is observed that an intriguing distribution of machine learning models were implemented in the examined studies. The predominant model of choice was the Neural Network (NN), utilized in a total of 10 studies, illustrating a preference for its ability to model complex non-linear relationships and its inherent aptitude for handling high-dimensional data typical in siRNA research. The Support Vector Machine (SVM) model was adopted in five studies, reflecting its well-regarded robustness and efficacy in dealing with both linear and non-linear classification problems. Meanwhile, the Random Forest (RF) model was employed in three studies, underscoring its suitability for managing multi-dimensional datasets with its ensemble-based approach and inherent feature selection mechanism. Lastly, the Partial Least Squares (PLS) model was applied in two studies, illustrating its utilization in situations where predictors are highly correlated, a common occurrence in biological data. The majority of studies employed correlation and least squares as performance metrics in their analyses. Notably, some studies deployed multiple models, acknowledging the unique strengths of each model and adopting a more holistic, hybrid approach to tackle the multifaceted complexities inherent in siRNA design and analysis.

## 2 Machine learning methods for small interfering RNAs

### 2.1 Predictions of siRNA efficacy and off-target effects

Machine learning has emerged as a crucial tool in the field of siRNA research, facilitating nuanced investigations into the complex dynamics of siRNA. Two particularly salient areas of exploration are the prediction of siRNA efficacy and the elucidation of off-target effects. A comparative analysis of the studies by La Rosa et al. (2022) and Metwally et al. (2022) reveals distinct approaches to the application of machine learning methodologies for predicting siRNA efficacy.

La Rosa et al. (2022) implemented a novel Graph Neural Network (GNN) for evaluating siRNA-mRNA interaction networks, with an aim to predict siRNA efficacy. This approach marked a significant stride in the research area as GNNs, which outperformed conventional machine learning algorithms, were introduced for the first time in this context. Their method proved successful with a notable Pearson correlation coefficient of approximately 73.6%, representing the siRNA’s ability to bind and silence a gene target effectively.

On the other hand, Metwally et al. (2022) took a different approach by constructing a machine learning model for *in silico* prediction of siRNA ionizable-lipid nanoparticles’ *in vivo* efficacy. The authors adopted an array of machine learning techniques, including Artificial Neural Networks (ANNs) and SVM, for Quantitative Structure-Activity Relationship (QSAR) modeling, signifying a broader perspective of machine learning implementation. Notably, their model successfully predicted the siRNA dose, with ANNs delivering the most robust performance.

Exploring off-target effects and RNA production regulation, Müllerr et al. (2021) utilized supervised machine learning to adjust for variables that indirectly influence global RNA production in HeLa cells. This work provides an extensive dataset that paves the way for future exploration into global RNA metabolism regulation and its correlation with cellular states. Conversely, Kobayashi et al. (2022) focused on the off-target effects of siRNA, demonstrating that such effects are influenced by the base-pairing stability of two distinct regions with contrasting effects. Their thorough examination of siRNA’s subregions via an array of machine learning techniques established an important correlation between thermodynamic properties and off-target influence, thereby enhancing our understanding of siRNA’s off-target effects.

Both the methodological approaches, i.e., GNN and ANNs/SVM, show significant potential in siRNA research, albeit in different contexts. While GNN exhibited a superior ability in siRNA-mRNA interaction analysis, the versatility of ANNs/SVM was beneficial in predicting *in vivo* efficacy of siRNA nanoparticles. Further, machine learning proved to be instrumental in understanding off-target effects and RNA production regulation, demonstrating the versatility and potential of these techniques in decoding the complexities of siRNA.

### 2.2 Unveiling cellular processes involving siRNA

The integration of high-throughput screening in siRNA studies has ushered in a new era of comprehensive insights into cellular behavior and mechanisms under the influence of genetic modification. These following articles underscore this trend, showcasing how machine learning techniques are employed to delve deeper into proteome regulation, cellular delivery, and phenotype expression.

Scott et al. (2020) and Kustatscher et al. (2023) provide perspectives on the use of machine learning in high-content screening and proteome regulation. Both studies underscore the effectiveness of machine learning in uncovering complex biological systems. Scott et al. (2020) used multiparameter principal component analysis and an unbiased, parameter-agnostic machine learning approach to uncover genes and pathways that regulate mitochondrial clearance. This approach allowed the exploration of siRNA-based screening data in detail and led to the identification of modulators of parkin recruitment to mitochondria.

On the other hand, Kustatscher et al. (2023) took a higher-level perspective, using machine learning to analyze proteomics data and discover co-regulation modules, termed “progulons”. This approach offered a robust framework for studying the human proteome, identifying 31 progulons that constitute core cellular functions. Supervised machine learning, in this case, not only facilitated data processing but also uncovered new replication factors. The comparison underscores the flexibility and utility of machine learning applications in different research settings and objectives, both achieving significant findings in their respective domains.

Two recent studies provided insights into the usage of machine learning for precision delivery and phenotype assessment in siRNA studies, respectively. Patino et al. (2022) demonstrated the use of a live-cell analysis device (LCAD) coupled with deep learning to perform localized electroporation-induced membrane permeabilization, allowing precise siRNA delivery and content extraction from live cells. The combination of deep learning with LCAD technology represents a synergistic integration of novel hardware and advanced analytical tools, suggesting new opportunities for precise genetic interventions and real-time cellular response monitoring. Eismann et al. (2020) introduced an automated screening workflow using light-sheet microscopy to evaluate mitotic phenotypes in 3D cell cultures following siRNA knockdown. They employed a convolutional neural network (CNN) for phenotype classification, achieving high-throughput screening with high spatiotemporal resolution. This methodology enables a precise assessment of mitotic phenotypes in an automated, high-throughput manner, highlighting the power of deep learning in image processing and phenotype recognition.

These studies underscore the significant potential of machine learning, from neural networks to principal component analysis, in advancing siRNA research. Whether it is the identification of new cellular pathways, the precise delivery of functional molecules, or the high-throughput screening of phenotypes, machine learning methodologies emerge as vital tools, showcasing the increasing intersection between computational and biological sciences.

### 2.3 Elucidating the role of siRNA in diseases

Biomedical research has witnessed the transformative potential of machine learning, catalyzing breakthroughs in disease diagnosis and prognosis. Two notable applications of these technologies involve the identification of prognostically significant genes in cancer and the discovery of diagnostic gene biomarkers. In these contexts, the incorporation of deep learning methods has yielded significant insights.

The study by Kukita et al. (2022) demonstrated the use of machine learning in combination with siRNA, chromatin immunoprecipitation sequencing, and RNA sequencing for pinpointing prognostically significant genes, focusing on endometrial cancer. The researchers identified that the histone methyltransferase SETD8 regulates gene expression via H4K20 methylation and the p53 signaling pathway. Interestingly, they observed that suppressing SETD8, through siRNA or a selective inhibitor, could potentially inhibit cell proliferation and instigate apoptosis in endometrial cancer cells. This example of machine learning implementation showcases the potency of these methods in generating meaningful and impactful discoveries in cancer research.

On the other hand, Sun et al. (2022) used machine learning to identify diagnostic gene markers associated with immune infiltration in patients with renal fibrosis. They integrated Support Vector Machine Recursive Feature Elimination (SVM-RFE) and Least Absolute Shrinkage and Selection Operator (LASSO) regression models to achieve this. Their study identified nine key genes, with the knockdown of ISG20 via siRNA significantly inhibiting renal fibrosis progression *in vitro*. This study is a compelling example of how machine learning can drive novel insights in diagnostic biomarker discovery and influence therapeutic strategies.

Both studies demonstrate the profound potential of machine learning in the exploration of disease genetics, either in a prognostic or diagnostic capacity. However, the approaches vary in their specificity. The approach by Kukita et al. (2022) primarily focused on the downstream effects of a specific gene (SETD8), whereas the method by Sun et al. (2022) was more general, analyzing a broader set of potential markers. Despite these differences, both studies effectively incorporated machine learning to inform and enrich our understanding of disease biology.

### 2.4 siRNA delivery and drug discovery

The convergence of deep learning and machine learning techniques is propelling siRNA research, particularly in designing efficient delivery systems and accelerating the drug discovery process. As exemplified in the study by Kavya et al. (2022), an Artificial Neural Network (ANN) model was utilized to predict the release behavior of drugs and genes from a curcumin-loaded polymer synthesized in supercritical CO_2_, encapsulating both curcumin and Bcl_2_ siRNA. The promising results obtained underscore the potential of deep learning models in predicting siRNA delivery and release patterns, thus potentially revolutionizing the design of more effective siRNA delivery systems.

On the other hand, Kuthuru et al. (2019) leveraged machine learning methodologies to predict drug-kinase-target interactions from a high-content analysis data from an siRNA human kinome screen. They developed two types of kinase descriptors and applied machine learning models to predict these interactions, with the top model achieving an area under the ROC curve of 0.86. This clearly indicates the potential of machine learning in expediting the process of drug discovery by accurately predicting drug-target interactions.

### 2.5 Other emerging topics in siRNA research

The rise of machine learning techniques has brought about a significant paradigm shift in siRNA research. Recent investigations have successfully harnessed both traditional machine learning and deep learning approaches to address challenges and answer pivotal questions in the field. This subsection provides the recent trend of emerging machine learning methodologies in siRNA studies, focusing on their unique applications, effectiveness, and particular roles in advancing siRNA research.

In relation to nanopore technology, Penguin (Hassan et al., 2022) and Sequoia (Koonchanok et al., 2023) have emerged as significant tools that leverage machine learning for direct RNA sequencing data analysis. Penguin is designed to identify pseudouridine sites in RNA, employing machine learning models such as SVM, RF, and NN to process the raw signal generated by Oxford Nanopore sequencing. On the other hand, Sequoia provides a comprehensive framework for visual analysis of RNA modifications from nanopore sequencing data, enabling users to interactively analyze and cluster signals based on electric-current similarities. In structural profiling of low molecular weight RNAs, Wang et al. (2021) proposed a novel machine learning algorithm to augment nanopore trapping/translocation. This algorithm transformed raw event characteristics into interpretable data, with an impressive accuracy of approximately 93.4%. Importantly, the algorithm was able to distinguish between various RNA types, demonstrating its potential for future siRNA studies. On a different note, He et al. (2019) applied a deep learning-based approach for predicting virus-derived small interfering RNAs (vsiRNAs) in plants. Their deep Convolutional Neural Network (CNN) model, PVsiRNAPred, trained on vsiRNA sequences, demonstrated superior performance to five conventional machine learning classifiers, achieving an accuracy of 65.70%. Both studies utilized machine learning, but their focus and approach differed, reflecting the versatility of machine learning applications in siRNA research.

Nademi et al. (2021) employed machine learning for predicting the cellular uptake of hydrophobically modified Polyethylenimine (PEI)/siRNA nanoparticles in various cancer cell lines. Using three regression models, the study revealed that non-linear models, such as RF and Multilayer Perceptron (MLP), outperformed the Linear Regression model in predictive accuracy. The predictive performance of these non-linear models shows their potential in improving our understanding of siRNA nanoparticle uptake in cancer research.

Meanwhile, Persson Hoden et al. (2021) developed an R package, smartPARE, that utilizes a deep learning CNN for the identification of true mRNA cleavage sites. Applied to high-throughput datasets, smartPARE effectively identified true cleavage sites, providing crucial insights into the small RNA (sRNA) landscape in complex biological systems.

In conclusion, the application of machine learning, from traditional algorithms to deep learning methods, is proving vital in various aspects of siRNA research, including structural profiling, prediction of vsiRNAs, cellular uptake prediction, and identification of mRNA cleavage sites. Although the methods and applications vary, the overall advancement in the field signifies the transformative potential of machine learning in this area. The comparison further highlights the benefits of non-linear and deep learning models over traditional linear models in terms of predictive accuracy and versatility, leading to valuable discoveries in the field of siRNA research.

## 3 Discussions: Future perspectives and challenges

The confluence of machine learning and siRNA research has already brought to light numerous applications and technological advancements. From predicting siRNA efficacy and off-target effects, to uncovering cellular processes involving siRNA and elucidating the role of siRNA in diseases, the combination of these fields has opened up new avenues for scientific exploration and innovation. However, similar to all emerging fields, it comes with its own set of unique challenges and limitations.

The application of machine learning techniques in siRNA research is hampered by the lack of expansive, high-fidelity datasets. This is a common problem in many machine learning applications, but it is particularly pronounced in the field of biological research, where experimental data can be costly and time-consuming to generate. However, as techniques such as high-throughput screening continue to advance, the availability of high-quality datasets for siRNA research is expected to increase. Moreover, strategies such as transfer learning and data augmentation could be leveraged to overcome the scarcity of data and enhance the learning capacity of machine learning models. As an alternative approach, the high-content screening techniques proposed by Scott et al. (2020) can be used to address this limitation of high-fidelity datasets.

Another significant challenge is the inherent complexity of biological systems. The multitude of interacting factors and the non-linear nature of biological processes pose significant difficulties for the construction and optimization of machine learning models. However, novel machine learning methods, such as GNN and deep learning (La Rosa et al., 2022), have demonstrated promising results in dealing with such complexities. Future work should continue to explore and optimize these techniques for application in siRNA research.

Finally, the lack of interpretable machine learning models in this field is a crucial area that needs to be addressed (Murdoch et al., 2019). Despite the promising results achieved by complex models such as deep learning, their black-box nature poses a significant challenge for their broader acceptance and utilization. Therefore, the development and application of more transparent, interpretable models should be a priority for future research.

While challenges and obstacles lie ahead, the potential rewards of integrating machine learning and siRNA research are vast. It is our hope that this review will provide a useful roadmap for future researchers navigating this exciting and rapidly evolving field.

## 4 Conclusion

In conclusion, the fusion of machine learning and siRNA marks a promising Frontier in the realm of RNA interference. Although faced with challenges, such as the need for large, high-quality datasets and the intricate nature of biological systems, the continued development of advanced machine learning models and feature engineering techniques offers an optimistic outlook on the field’s future.

The rapidly evolving landscape of machine learning necessitates frequent and thorough investigation of recent studies, particularly when coupled with the emergent field of siRNA. This review has thus aimed to provide a comprehensive, timely exploration of these two intertwined fields, bridging the gap between computational advancements and biological complexities. It is our fervent hope that this work will serve as a foundation for future explorations and will inspire novel, cross-disciplinary endeavors.
